# When and How to Perform Open Aortic Repair After Thoracic Endovascular Repair Complications?

**DOI:** 10.1016/j.ejvsvf.2025.01.004

**Published:** 2025-01-26

**Authors:** Paulo Eduardo Ocke Reis, Arindam Chaudhuri

**Affiliations:** a Universidade Federal Fluminense, Rio de Janeiro, Brazil; b Bedfordshire – Milton Keynes Vascular Centre, Bedfordshire Hospitals NHS Foundation Trust, Bedford, UK

**Keywords:** Aortic surgery, TEVAR aortic, TEVAR aortic dissection, TEVAR aortic complication, Vascular surgery, Endovascular surgery

Endovascular treatment of aortic aneurysms or dissections compared with open surgery is less invasive and affords lower morbidity and mortality rates. The aim of thoracic endovascular aortic repair (TEVAR) is to achieve complete exclusion of the aneurysm sac using prostheses from within with typically few complications. However, adverse events can occur, such as infection of the prosthesis, persistence of blood flow in the aneurysmal sac (or false lumen as relevant for interventions for aortic dissection) after endovascular interventions. Although it is difficult to assess the global rate of open aortic repairs after TEVAR, the conversion rates in large series of TEVARs undertaken (*n* > 300) have ranged from 0.4 to 7.9 % as indicated by Coselli *et al.*^1^

The current paper presented by Iba *et al.*^2^ raises questions including what parameters guide the need and scope for an open salvage approach, and when to undertake conversion for endoleaks or any other complication, a salient problem being infection. This small but relevant series presents cause specific mortality rates and importantly indicates that there are no data particularly around the issue of criteria or protocols for open conversion after complications such as infection, considering that aorto-enteric fistula (AEF) in this scenario had very high mortality rate,^2^ though we commonly consider that the typical indications identified for open re-intervention are endoleak and further aneurysmal degeneration. They further indicate a range of salvage interventions are needed including arch reconstruction and thoracic aortic replacement, besides management of the oesophagus in the AEF scenario, intercostal artery ligation as a targeted intervention for endoleakage, with reasonable overall results including adverse outcome rates for stroke and paraplegia.

Hybrid arch repair has been used with one of a number of open surgical procedures together with placement of an endograft in the arch or descending aorta, when associated with descending aortic aneurysms, the frozen elephant trunk technique is used followed by stent grafting of the descending thoracic aorta, it is denoted minimally invasive because it avoids aortic cross clamping, blood loss, hospital stay, and hypothermic circulatory arrest; however, the morbidity and mortality rates remain considerably high: mortality 0–15%, stroke 0–11%.^3^^,^^4^ Linked to this, at the outset, a totally endovascular treatment of aortic arch pathology was difficult because of the supra-aortic vessels.^3^^,^^4^ Development of endograft technology has enabled total endovascular repair of complex aortic aneurysms, using fenestrated and branched endografts or with the sandwich technique,^3^, ^4^, ^5^ which may then also influence the complexity of open salvage repairs if needed. It is important to highlight that in some reports the complication rate is higher following TEVAR, and is estimated to be as high as 38% and that common complications include both those related to the endograft device and systemic complications.^6^ Device related complications include endoleaks, endograft migration or collapse, kinking and or stenosis of an endograft limb, and graft infection.^4^, ^5^, ^6^ Post-procedural systemic complications include end organ ischaemia, cerebrovascular and cardiovascular events and post-implantation syndrome. Secondary re-interventions are required in approximately 19–24% of cases following endovascular abdominal as well as thoracic aortic aneurysm repair, respectively.^6^

As the devices available for endovascular repair evolve, and new devices emerge in the endovascular market expanding the scope of TEVAR, ongoing review of these outcomes will be necessary. It will thus also be important to capture and analyse trends relating to such results as further techniques become available for endovascular repair of the ascending aorta and aortic arch.^7^ Overall, the paper indicates that we need more robust data and thus evidence around the subject of post-TEVAR open conversion in the future, particularly as procedural scope expands, and numbers increase with possibly more post-TEVAR cases needing complex explantation and open repair.^8^ This may come through device capture in registries,^9^ including perhaps assessment of antibiotic prophylaxis aspects as a starting point, but will need to then also necessarily factor in long term follow through, which remains a shortcoming in the data capture process. The authors also demonstrate possibly acceptable results after such high risk complex re-interventions, with the subtle indication that these are best undertaken in cardiothoracic centres with specialist aortic operators.
